# Automated Scoring of Chromogenic Media for Detection of Methicillin-Resistant Staphylococcus aureus by Use of WASPLab Image Analysis Software

**DOI:** 10.1128/JCM.02778-15

**Published:** 2016-02-25

**Authors:** Matthew L. Faron, Blake W. Buchan, Chiara Vismara, Carla Lacchini, Alessandra Bielli, Giovanni Gesu, Theo Liebregts, Anita van Bree, Arjan Jansz, Genevieve Soucy, John Korver, Nathan A. Ledeboer

**Affiliations:** a Medical College of Wisconsin, Milwaukee, Wisconsin, USA; b Wisconsin Diagnostic Laboratories, Milwaukee, Wisconsin, USA; c A. O. Ospedale Niguarda Cà Granda, Milan, Italy; d PAMM Laboratory of Medical Microbiology, Veldhoven, Netherlands; e CHU de Quebec-Universite Laval, Quebec City, Quebec, Canada; f Hamilton General Hospital, Hamilton, Ontario, Canada

## Abstract

Recently, systems have been developed to create total laboratory automation for clinical microbiology. These systems allow for the automation of specimen processing, specimen incubation, and imaging of bacterial growth. In this study, we used the WASPLab to validate software that discriminates and segregates positive and negative chromogenic methicillin-resistant Staphylococcus aureus (MRSA) plates by recognition of pigmented colonies. A total of 57,690 swabs submitted for MRSA screening were enrolled in the study. Four sites enrolled specimens following their standard of care. Chromogenic agar used at these sites included MRSASelect (Bio-Rad Laboratories, Redmond, WA), chromID MRSA (bioMérieux, Marcy l'Etoile, France), and CHROMagar MRSA (BD Diagnostics, Sparks, MD). Specimens were plated and incubated using the WASPLab. The digital camera took images at 0 and 16 to 24 h and the WASPLab software determined the presence of positive colonies based on a hue, saturation, and value (HSV) score. If the HSV score fell within a defined threshold, the plate was called positive. The performance of the digital analysis was compared to manual reading. Overall, the digital software had a sensitivity of 100% and a specificity of 90.7% with the specificity ranging between 90.0 and 96.0 across all sites. The results were similar using the three different agars with a sensitivity of 100% and specificity ranging between 90.7 and 92.4%. These data demonstrate that automated digital analysis can be used to accurately sort positive from negative chromogenic agar cultures regardless of the pigmentation produced.

## INTRODUCTION

Automation in clinical chemistry and hematology laboratories has been widely available for years, but only recently have these changes been adapted for clinical microbiology. The initial advances in automation of the microbiology lab include continuously monitored blood cultures and mycobacterial growth and automated antimicrobial susceptibility testing systems. Numerous studies have demonstrated the benefit of these systems in reducing turnaround time (TAT), reducing labor costs, and improving patient care (1–4). The success and impact of these systems have opened the door to further automation, including the processing of microbial specimens. Similar to results seem with incorporation of automation in other parts of the laboratory, studies have demonstrated that incorporation of automated specimen processors can improve patient care by producing more isolated colonies than manual plating, reducing laboratory costs, and reducing plate contamination (5–7).

Manufacturers have improved on previous specimen processors by adding conveyor/track systems to move plates into incubators, programmable software to adapt to various laboratory protocols, and digital cameras, which can be accessed at workstations using a computer and high-definition monitor, to image plates at various time points. The goal of these improvements is to create full laboratory automation systems that process specimens, incubate plates, image plates for interpretation, and pick colonies for further culture workup. To date, the Kiestra total laboratory automation (BD Kiestra B.V., Drachten, Netherlands) and the WASPLab (Copan, Brescia, Italy) systems have been marketed to clinical laboratories and include several of the above features. Although the technology may not yet be able to identify organisms based on colony morphology, digital imaging can currently identify the presence of colonies on a plate and distinguish between different colors, such as those found on chromogenic agars.

Chromogenic agars are specific media that take advantage of the differences in pathogen metabolism by creating enzymatic reactions specific for target organisms, such as vancomycin-resistant enterococci (VRE), group B streptococcus (GBS), and methicillin-resistant Staphylococcus aureus (MRSA) (8–10). When the target is present, substrates produced during growth interact with the chromogen to produce pigmentation (mauve, pink, or green). With digital imaging software capable of distinguishing differences in pixel color, chromogenic agar is ideal for digital automation as color thresholds can be created to detect target growth.

**FIG 1:**
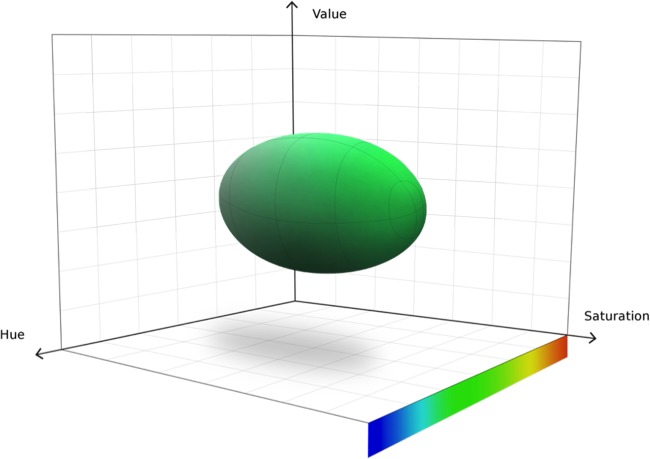
HSV color space, where H (hue) represents the type of color, S (saturation) represents the intensity of the color, and V (value) represents the brightness of the color. The “bubble” is the visual representation of the threshold volume in this three-dimensional space.

The WASPLab chromogenic detection module (CDM) is software that analyzes digital images for a customizable target color by converting red-green-blue (RGB) images into a 3-dimensional space composed of hue, saturation, and value (HSV), creating a “bubble-shaped” tolerance level for defining “nonnegative” media plates. Figure 1 demonstrates a bubble as the target definition space. To detect nonnegative/negative plates, the software analyzes every pixel (each medium plate image is composed of 27 million pixels) in the image, looking for the selected color pattern within the specified tolerance. Plates containing pixels with HSV values within the set parameters are marked as nonnegative, whereas plates are marked negative if no pixel contains an HSV score outside the parameters. We hypothesize that implementation of the CDM software into the WASPLab can accurately sort chromogenic MRSA plates as nonnegative or negative.

To evaluate the performance of the CDM software, we performed a multisite evaluation of the WASPLab to identify MRSA from swab cultures plated to various chromogenic agars. Four sites enrolled a total of 57,690 swabs that were collected for MRSA screening. Swabs were automatically plated by the WASPLab to chromogenic agar, and images were read by the CDM software and compared to a manual reading for detection of positive MRSA plates. To demonstrate the robustness and accuracy of the CDM software, 3 different chromogenic media were tested: MRSASelect (Bio-Rad Laboratories, Redmond, WA), chromID MRSA (bioMérieux, Marcy l'Etoile, France), and BD CHROMagar MRSA (BD Diagnostic, Sparks, MD). The CDM software threshold is set for each manufacturer's agar, as pigmentation varies between plates (pink for MRSASelect, green for ChromID MRSA, and mauve for CHROMagar MRSA).

## MATERIALS AND METHODS

### Specimen processing.

Four laboratories from various geographical locations were involved in this study. These sites included A. O. Ospendale Niguarada (Milan, Italy), PAMM laboratories (Veldhoven, Netherlands), CHU de Quebec (Quebec City, Canada), and Hamilton General Hospital (Hamilton, ON, Canada). All 4 laboratories involved in this study routinely perform MRSA screens using ESwabs (Copan, Brescia, IT and Murrieta, CA, USA) to collect specimens from anterior nares, throat, perineum, or open wounds, which are then plated onto chromogenic agar. For this study, ESwabs received by the laboratory were enrolled in the study and tested according to the laboratory standards of care. Briefly, swabs were loaded into the WASPLab for plating on chromogenic agar. One site performed an enrichment step using nutrient broth (10 g/liter Lab Lemco powder, 10 g/liter peptone, and 5.0 g/liter NaCl) (Oxoid, Basingstoke, United Kingdom), which was incubated for 18 to 24 h at 37°C prior to plating. Once plated, the WASPLab transferred the plate to the WASPLab incubator where the on-board camera collected a time point 0 image. The plates were then incubated at 35 to 37°C for 16 to 24 h, depending on the laboratory standard operating procedures and manufacturer's instructions for use. After the established incubation period, a second image was collected, saved, and used for both automated and manual reading. Approval by each site's institutional review board or oversight committee was obtained prior to any specimen enrollment.

### Automated digital analysis of chromogenic media.

The chromogenic detection module (CDM) image analysis software scans the image of the surface of the plate, looking for colored pixels; growth is identified by a comparison to the time point 0 plate. Depending on the chromogenic plate used (green, pink, or mauve colonies), an HSV threshold that reported plates as nonnegative or negative for MRSA was set. In this study, colonies containing HSV values that fell within the tolerance threshold were reported as automation positive (AP). In the absence of typically colored colonies, the specimen was reported as automation negative (AN).

### Manual reading of chromogenic plates.

Technologists reading plates manually were blinded to the results with the software. After 16 to 24 h of incubation, a technologist individually reviewed each plate's digital image (the same image used for the automated analysis). Depending on the chromogenic medium used by the laboratory, the technologist looked for colonies containing the color indicated in the package insert (pink for MRSASelect, green for ChromID MRSA, mauve for CHROMagar MRSA). Each plate was scored as manual positive (MP) or manual negative (MN) by the technologist based on the presence of indicated colonies. Colonies that technologists identified as questionable (hue differences) were removed from the incubator, and a Gram stain and catalase and latex agglutination tests were performed to further determine the presence of S. aureus.

### Discrepant analysis.

Data analysis was performed retrospectively, so discrepant specimens were not available for further workup. To reconcile these discrepant specimens, the digital images were sent back to the corresponding laboratories to be reviewed by a supervisor or the laboratory director. Each image was reviewed, and all discordant results were reported as having either excess matrix background creating pigmentation of the agar (residual matrix), a borderline colony color that would not be worked up by the laboratory (borderline colors), or plates where the technologist missed a colony (automation-positive 2nd manual-positive results).

### Statistical analysis.

Results from the software's digital analysis were compared to the technologist's manual reading as the true value. The performance characteristics, including sensitivity and specificity, were calculated using standard methods. The 95% confidence intervals (CIs) were calculated by using a binomial expansion.

## RESULTS

### Comparison of automatic imaging to manual detection of MRSA-positive chromogenic agar.

**FIG 2:**
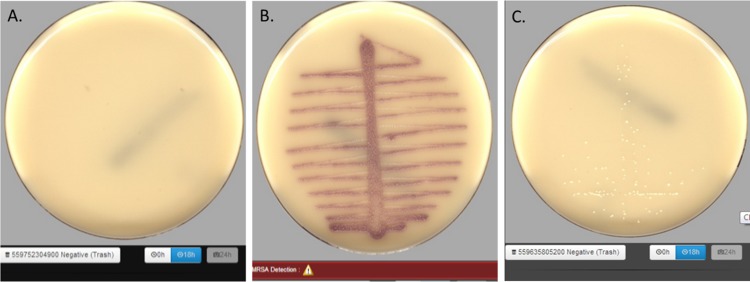
Representative examples of chromogenic media generated by WASPLab imaging. (A) Negative chromogenic plate containing no growth; (B) positive chromogenic plate containing MRSA; (C) a chromogenic plate with non-MRSA growth, small white colonies.

The image taken by the onboard camera is a composite image that uses several light sources and several lighting intensities to simulate manual reading of a plate. Representative images of plates with no growth, plates positive for MRSA, and plates with growth lacking pigmentation are shown in Fig. 2. Technologists performing manual interpretation used similar images to determine if the plates were positive for MRSA.

In total, 57,690 swabs were enrolled and tested at 4 different locations. The overall prevalence of MRSA was observed to be 2.4% and ranged from 2.1 to 7.3% at the testing sites. Of the 57,690 plates analyzed, 1,367 plates were called positive for MRSA by both automation and manual reading (Table 1). An additional 5,210 (9.0%) plates that had been manually read as negative were reported as nonnegative by the CDM software. Importantly, the automatic imaging software did not read any manual-positive plates as negative. Together these data demonstrated an overall sensitivity of 100% (95% CI, 99 to 100%) and a specificity of 90.7% (95% CI, 90 to 91%). Data were similar across all four sites with specificities ranging from 90.0 to 96.0%.

**TABLE 1:** Performance of WASPLab digital imaging compared to manual reading

Clinical test site	No. of specimens tested	Results (no.)^*a*^				Performance (% [95% CI])^*b*^	
		MP/AP	MN/AN	MN/AP	MP/AN	Sensitivity	Specificity
1	5,604	119	5,266	219	0	100 (96–100)	96.0 (95–96)
2	41,064	680	36,333	4,051	0	100 (99–100)	90.0 (89–90)
3	2,217	162	1,898	157	0	100 (97–100)	92.4 (91–93)
4	8,805	406	7,616	783	0	100 (99–100)	90.7 (90–91)
Total	57,690	1,367	51,113	5,210	0	100 (99–100)	90.7 (90–91)

a MP/AP, manual positive/automation positive; MN/AN, manual negative/automation negative; MN/AP, manual negative/automation positive; MP/AN, manual positive/automation negative.

b CI, confidence interval.

### Analysis of manual-negative/automation-positive plates.

**FIG 3:**
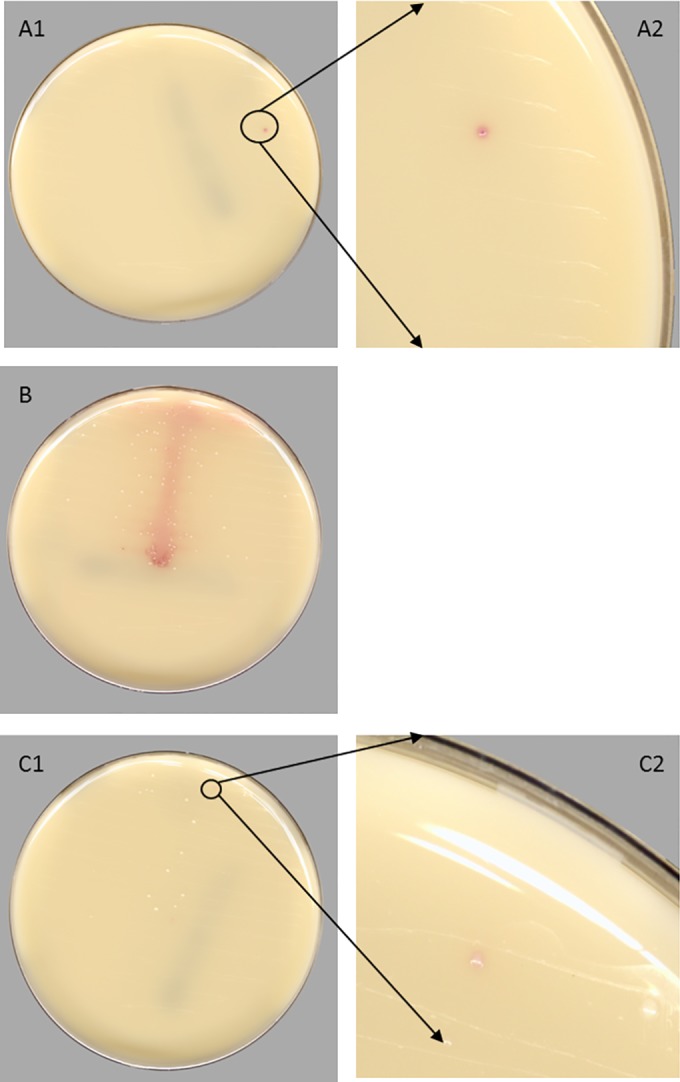
Representative examples of manual-negative automation-positive plates generated by WASPLab CDM software. (A1 and A2) Automation-positive 2nd manual-positive plate showing a small colony not visually detected by manual examination but accurately identified as positive by the CDM. (B) Residual matrix on the plate showing lack of growth, but containing color due to the presence of specimen matrix. (C1 and C2) Borderline color plate demonstrating similar color colonies.

In an effort to reduce the false-negative results, the threshold bubble was large for all testing. Use of a conservative threshold resulted in a manual-negative/automation-positive (MN/AP) rate of 9.0% (5,210/57,690). Reexamination of these MN/AP plates by a supervisor or laboratory director identified three different types of discrepancies, which we have categorized as (i) automation-positive 2nd manual-positive result, (ii) residual matrix, or (iii) borderline colors. An example of each of these categories is shown in Fig. 3. Automation-positive 2nd manual-positive results were the least common, representing 2.9% (153/5,210) of the discrepant results found in this study (Table 2). These results are defined as small colonies that were not visually detected by the initial manual examination but upon review should have been called positive by the laboratory, suggesting that the CDM software was correct. Residual matrix represented 22.8% (1,189/5,210) of the discrepant specimens and comprised plates containing colorimetric agar not associated with microbial growth. The most common discrepancy was borderline colors, where the CDM software calculated scores within the threshold, but manual examination did not detect any positive color. This class represented 74.2% (3,868/5,210) of the discrepant results, due to the conservative setting of the threshold designed to prevent false-negative results.

**TABLE 2:** Discrepant analysis of manual negative/automation positive plates

Discrepant category	No. of plates
MN/AP^*a*^	5,210
Automation positive 2nd manual positive	153
Residual matrix	1,189
Borderline colors	3,868

a Manual negative/automation positive.

### Comparison of 3 chromogenic media for the detection of MRSA from swabs.

The sites participating in the study used only the chromogenic agar outlined in their standard of care, and not all sites used the same chromogenic agar. Agars used in this study were MRSASelect (Bio-Rad Laboratories, Redmond, WA), chromID MRSA (bioMérieux, Marcy l'Etoile, France), and BD CHROMagar MRSA (BD Diagnostic, Sparks, MD). The sensitivities for all of these chromogenic agars were equivalent when the CDM software was used (Table 3). The specificities for the three chromogenic agars were 90.7% (MRSASelect), 92.4% (chromID), and 90.7% (BD CHROMagar).

**TABLE 3:** Comparison of 3 chromogenic agars for the detection of MRSA

Chromogenic medium	No. of specimens tested	Results (no.)^*a*^				Performance (% [95% CI])^*b*^	
		MP/AP	MN/AN	MN/AP	MP/AN	Sensitivity	Specificity
MRSASelect	46,668	799	41,599	4,270	0	100 (99–100)	90.7 (90–91)
chromID MRSA	2,217	162	1,898	157	0	100 (97–100)	92.4 (91–93)
BD Chromagar MRSA	8,805	406	7,616	783	0	100 (99–100)	90.7 (90–91)

a MP/AP, manual positive/automation positive; MN/AN, manual negative/automation negative; MN/AP, manual negative/automation positive; MP/AN, manual positive/automation negative.

b CI, confidence interval.

## DISCUSSION

To date there have been limited studies demonstrating the benefits for implementation of full laboratory automation in clinical microbiology laboratories. A recent report observed an increase of approximately 2-fold in the laboratory production index (number of samples/staff members/day) when full lab automation is used (5). Although data demonstrating improvements in efficiency associated with full laboratory automation are limited, further studies documenting efficiency are needed.

Currently, laboratories that are performing MRSA screening receive specimens throughout the day and manually plate each specimen to chromogenic agar. These plates are then incubated for 18 to 24 h; however, in practice this time can vary based on available staff and operation hours. After incubation, each plate is observed by a technologist and reported as positive or negative with staff reading hundreds of plates a day. In a laboratory incorporating the WASPLab into the workflow, the technologist loads specimens into the instrument and the instrument processes, tracks, incubates, and images the specimen and separate plates as nonnegative and negative. Twenty-four hours later, a technologist interfaces with a WASPLab workstation to perform analysis of specimens. When negative plates are imaged, up to 40 plates can be observed on the screen at one time, confirmed negative, and discarded with a single click. Although for this study each plate was viewed individually, this workflow would have reduced the amount of screen images viewed by a technologist from 51,113 to 1,278 (negative plates/40 images per screen). Nonnegative plates are called up individually, and the technologist can score these similarly to the previous workflow, but without the need to physically obtain the plate. Quick removal of negative plates will ease the burden of large-volume screens.

The automation of digital imaging might also help laboratory workflow as plates are always imaged within the appropriate time frame, potentially reducing turnaround time. Analyzing the chromogenic media at 16 to 24 h is important because specificity is lost (from breakthrough growth and degradation of products) as the plate incubates beyond the recommended duration. Joubrel et al. observed that the specificity for detection of Salmonella on chromogenic agar decreased as the incubation periods increased from 24 to 48 h. The specificity was reported as 91% at 24 h and was reduced to 84% at 48 h postinoculation (11), which is consistent with the results of other studies evaluating various chromogenic media (12–14). Laboratories in which plate reading is delayed may overcall the readings for chromogenic media, resulting in overtreatment of patients. Implementation of CDM software would allow the technologist to review the plate as if it was read at 24 h, ensuring optimal specificity on the chromogenic agar. In addition, laboratories that cannot support testing over the weekend could allow screens to be ordered on Friday and reported on Monday without loss of specificity.

This is the first high-volume, multisite study demonstrating the ability of full lab automation to perform image analysis on different chromogenic media. For this study, thresholds in the WASPLab CDM software were set to ensure that any true positive was detected by the imaging software. Discrepant resolution demonstrated that the software overreported positive results due to minor pigmentation that is not associated with positive specimens or pigmentation of the agar due to residual matrix. These specimens are easily identifiable on a monitor and can be reported as negative by the technologist. No false-negative plates were identified during this study, demonstrating that the conservative thresholds set allowed the CDM software to be highly sensitive. Interestingly, the CDM software identified 153 specimens that were positive after a second review of the digital image. These data suggest that the CDM software may be more sensitive than manual observation.

The comparison of the chromogenic agars was not a direct comparison because we did not evaluate all media types at all sites, which is a limitation of this study. All media types in this study had similar sensitivity and specificity; however, as each specimen was only tested on one medium, testing was not a direct specimen-to-specimen comparison. The specimen enrollment was high at all sites, and no differences were observed, suggesting that specimen variability did not affect outcomes. In addition, this study was designed to blind the technologist from the software's results to remove bias, which is essential as the use of chromogenic plates is reliant on the technologist's judgment of growth and color. Data analysis was performed after all testing was completed, removing the ability of the laboratory to perform confirmatory testing. Because of this limitation, discordant results that contained either borderline colors or automation-positive 2nd manual-positive colonies might not be classified as MRSA or pigmented breakthrough growth.

The findings from this study demonstrated that automation can accurately remove negative plates and identify plates that can be misread by manual observation. Currently, the software cannot be used without technologist support as 5,057 false-positive results were reported. However, segregating 88.6% of the chromogenic plates will reduce the time and costs for clinical laboratories performing high-volume screens. Studies measuring TAT, patient outcomes, and cost analysis will help aid clinical directors in determining the utility of automated digital analysis.
